# Chrysophanol Suppresses Cell Growth via mTOR/PPAR-α Regulation and ROS Accumulation in Cultured Human Tongue Squamous Carcinoma SAS Cells

**DOI:** 10.3390/cimb44040104

**Published:** 2022-04-01

**Authors:** Po-Chih Hsu, Chia-Chen Hsu, Yi-Jan Hsia, Chan-Yen Kuo

**Affiliations:** 1 Department of Dentistry, Taipei Tzu Chi Hospital, Buddhist Tzu Chi Medical Foundation, New Taipei City 231, Taiwan; pino0906@gmail.com (P.-C.H.); because0517@gmail.com (C.-C.H.); yjhsia@tzuchi.com.tw (Y.-J.H.); 2 Graduate Institute of Medical Sciences, National Defense Medical Center, Taipei 114, Taiwan; 3 Department of Research, Taipei Tzu Chi Hospital, Buddhist Tzu Chi Medical Foundation, New Taipei City 231, Taiwan

**Keywords:** chrysophanol, mammalian target of rapamycin (mTOR), peroxisome proliferator-activated receptor-alpha (PPAR-α), reactive oxygen species (ROS), oral squamous cell carcinoma (OSCC)

## Abstract

Oral cancer, a type of head and neck cancer, can pose a significant risk of death unless diagnosed and treated early. Alternative treatments are urgently needed owing to the high mortality rate, limitations of conventional treatments, and many complications. The anthraquinone compound chrysophanol acts as a tumor suppressor on some types of cancer cells. To date, it has not been clarified how chrysophanol affects human tongue squamous carcinoma. This study was aimed to examine the effects of chrysophanol on oral cancer treatment. The results show that chrysophanol caused cell death, reduced the expression of the mammalian target of rapamycin (mTOR)/peroxisome proliferator-activated receptor-alpha (PPAR-α), and increased reactive oxygen species (ROS) production. We also used two ion chelators, deferoxamine (DFO) and liproxstatin-1 (Lipro), to further determine whether chrysophanol inhibits cell growth and regulates mTOR/PPAR-α expression and ROS production, both of which are involved in iron homeostasis. The results show that DFO and Lipro reversed the increase in cell death, downregulation of mTOR/PPAR-α, and decrease in ROS accumulation. In conclusion, chrysophanol inhibits the growth of oral squamous cell carcinoma cells by modulating mTOR/PPAR-α and by causing ROS accumulation.

## 1. Introduction

Several factors determine the geographic distribution of oral cancer, one of the top 10 cancers worldwide [1,2]. It is estimated that 76% of patients with intraoral cancer survive without metastasis after 5 years. Several epidemiological studies have identified risk factors for oral cancer, including tobacco use, eating betel nuts, excessive alcohol consumption, poor oral hygiene, inappropriate dentures, and other rough surfaces that irritate the mouth [3,4]. Approximately 90% of all oral cancers are oral squamous cell carcinoma (OSCC), which is more often found in men, with the risk increasing with age [5]. A large majority of OSCC cases occur on the tongue, which has little effect on other parts of the mouth [6]. At present, how oral squamous cell carcinoma develops remains controversial, thus compromising treatment and prognosis.

Chrysophanol, which was discovered in plants of the genus *Rheum*, is one of the most important anthraquinone components [7]. Chrysophanol has been reported to have anti-inflammatory, anti-cancer, and anti-depressive effects and offers neuroprotection [7,8]. In our previous study, chrysophanol was shown to be involved in the death, metastasis, and production of reactive oxygen species (ROS) in oral cancer cells [9] and to alter the Wnt-3-dependent signaling pathway, increasing epithelial–mesenchymal transition (EMT) formation, ROS accumulation, and metastasis [10]. We also suggest that migration/invasion and EMT are regulated by interleukin-6 and interleukin-8 in chrysophanol-treated oral cancer cells [11]. Thus, chrysophanol may disrupt the development of oral cancer. However, there is still some uncertainty regarding the protective role of chrysophanol against oral cancer.

Numerous studies have suggested that the mammalian target of rapamycin (mTOR) pathway is intrinsically linked to oral carcinogenesis and adverse prognosis [12,13,14]. Targeting mTOR has emerged as a promising therapy against OSCC and head and neck cancers [15]. PPAR-α is a nuclear hormone receptor that modulates the progression of oral cancer by influencing the behavior of one or more of its pathological features [16]. Therefore, targeting PPAR-α for oral cancer treatment might be crucial. In this study, we attempted to provide a theoretical basis for cancer treatment by exploring the effect of chrysophanol on OSCC via the regulation of mTOR and PPAR-α.

## 2. Materials and Methods

### 2.1. Reagents and Antibodies

Chrysophanol was obtained from Sigma-Aldrich (St. Louis, MO, USA). Western blotting was performed using antibodies against mTOR, PPAR-α, and GAPDH (ABclonal, Woburn, MA, USA).

### 2.2. Cell Culture

The SAS cell line (human tongue squamous carcinoma) was obtained from the National Defense Medical Center. Mycoplasma was not detected in any of the cells by DAPI staining 4 times for 1 year, according to a previous study [17]. The cell line was grown in Dulbecco’s Modified Eagle Medium (Gibco, New York, NY, USA) containing 10% fetal bovine serum (Gibco, Grand Island, NY, USA) and 1% penicillin/streptomycin (Gibco, Grand Island, NY, USA), and incubated at 37 °C with 5% CO_2_.

### 2.3. Cell Viability Assay

WST-1 (Roche Applied Sciences, Mannheim, Germany) was used to measure cell viability as previously described [18]. Briefly, seeded cells were plated in 24-well plates at a density of 5 × 10^4^ cells/mL and cultured 24 h later in DMEM containing 0.5% heat-inactivated FBS. Chrysophanol was then added to cells in indicated concentrations for 24 h. A reagent for WST-1 was added to the medium, and incubation was continued for 2 h at 37 °C. Finally, a microplate reader (Thermo LabSystems, Philadelphia, PA, USA) was used to measure absorbance at 450 nm.

### 2.4. Western Blotting

Cells were harvested and lysed according to the procedure described in our previous study [19]. Ice-cold RIPA lysis buffer (Sigma-Aldrich, St. Louis, MO, USA) was used to dissolve the pelleted cells. Cell lysates were obtained by centrifuging samples at 14,000× *g* for 20 min at 4 °C. Sodium dodecyl sulfate–polyacrylamide gel electrophoresis (SDS-PAGE) was used to separate proteins, followed by electrophoresis onto a nitrocellulose membrane. Primary and secondary antibodies (Cell Signaling Technology, Danvers, MA, USA) conjugated to horseradish peroxidase were used for immunoblotting. ECL kits (Millipore, Temecula, CA, USA) were used to measure protein expression, and images of the indicated proteins were subsequently analyzed using the ChemiDoc^TM^ XRS + System (Bio-Rad Laboratories, Hercules, CA, USA).

### 2.5. Measuring Intracellular ROS Generation

We measured ROS generation within 1 × 10^4^ cells by utilizing 10 μM 2′, 7′-dichlorofluorescin diacetate reagent (Sigma-Aldrich, St. Louis, MO, USA) for 30 min at 37 °C. Flow cytometry (FACScan, Becton Dickinson, Franklin Lakes, NJ, USA) was used to measure the ROS levels.

### 2.6. In Vivo Mice Xenografts

Female Nod.CB17-Prkdcscid/JNarl (NOD SCID) mice, aged 4–5 weeks, were purchased from BioLASCO Taiwan Co., Taipei, Taiwan. SAS cells (1.5 × 10^6^) mixed 1:1 in Matrigel (BD Biosciences, East Rutherford, NJ, USA) with phosphate-buffered saline (PBS) were injected subcutaneously into the flanks of the NOD SCID mice. The mice were treated with 1.67 mg/kg 3 times/week [7], and PBS was used for the control group. All mouse experiments were performed according to the guidelines of the Care and Use of Laboratory Animals, Taipei Tzu Chi Hospital, Taiwan, and were approved by the hospital’s Institutional Animal Care and Use Committee (Approval NO, 110-IACUC-014).

### 2.7. Statistical Analysis

Three triplicate samples were analyzed for each independent biological sample, and the average and standard deviation were calculated. Analyses of the data were conducted using Prism software (version 8.0; GraphPad Software). For continuous variables, ANOVA was used with a Bonferroni post hoc test to determine statistical differences between groups. Differences were considered statistically significant when the *p*-value was less than 0.05.

## 3. Results

### 3.1. Chrysophanol Causes Cell Death, Downregulates mTOR/PPAR-α, and Increases ROS Accumulation

**Figure 1:**
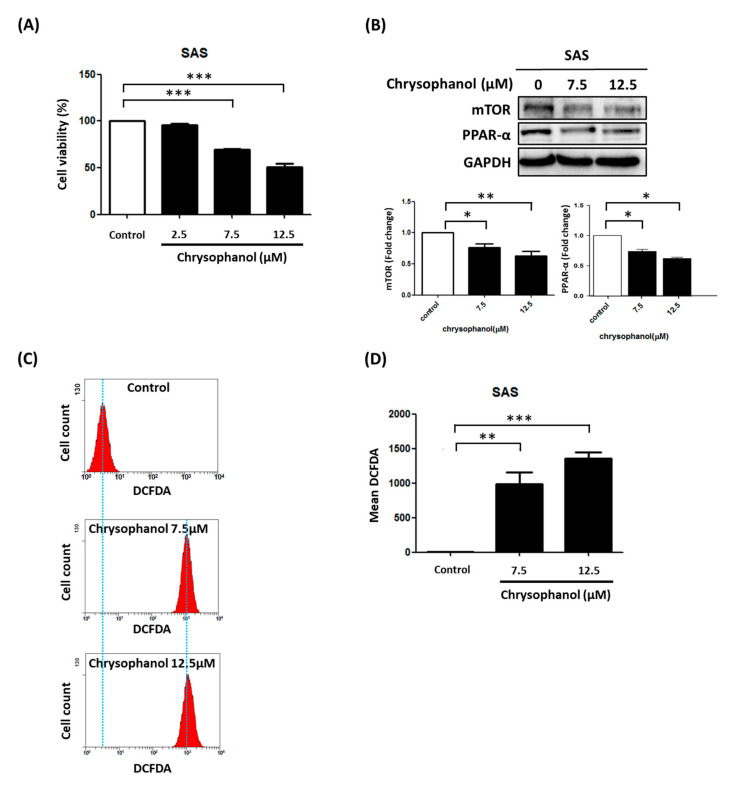
Effect of chrysophanol on cell survival, mTOR/PPAR-α expression, and ROS production in SAS cells. (**A**) WST-1 was used to measure viability of cells in indicated groups. (**B**) Upper panel indicates expression changes of mTOR and PPAR-α proteins in the presence of 0, 7.5, and 12.5 μM chrysophanol. Lower panel indicates quantitative results of assessing specific proteins using ImageJ. (**C**) Changes in ROS levels (with 10 μM DEFDA staining) with 0 (control), 7.5, and 12.5 μM chrysophanol treatment. (**D**) Mean fluorescence intensity was used to measure lipid ROS levels. Three independent replicates were used to calculate standard deviation; *n* = 3. * *p* < 0.05; ** *p* < 0.01; *** *p* < 0.001.

To determine whether chrysophanol causes SAS cell death, cell viability was evaluated after exposure to chrysophanol at different concentrations (0, 2.5, 7.5, and 12.5 μM) for 24 h. The results show that chrysophanol inhibited cell viability in a concentration-dependent manner (Figure 1A). We also verified the effect of chrysophanol on mTOR and PPAR-α expression using Western blotting. The results indicate that chrysophanol downregulated the expression of mTOR and PPAR-α in a concentration-dependent manner (Figure 1B). Accumulating evidence indicates that cancer treatment involves strategies that modulate ROS levels [20,21]. In our experiment, a significant increase in ROS production was observed in SAS cells after treatment with 7.5 and 12.5 μM chrysophanol (Figure 1C,D). These findings demonstrate that chrysophanol caused cell death, downregulated mTOR/PPAR-α, and increased ROS accumulation in SAS cells.

### 3.2. Chrysophanol Regulates Cell Death, mTOR/PPAR-α Expression, and ROS Accumulation in an Iron-Dependent Manner

**Figure 2:**
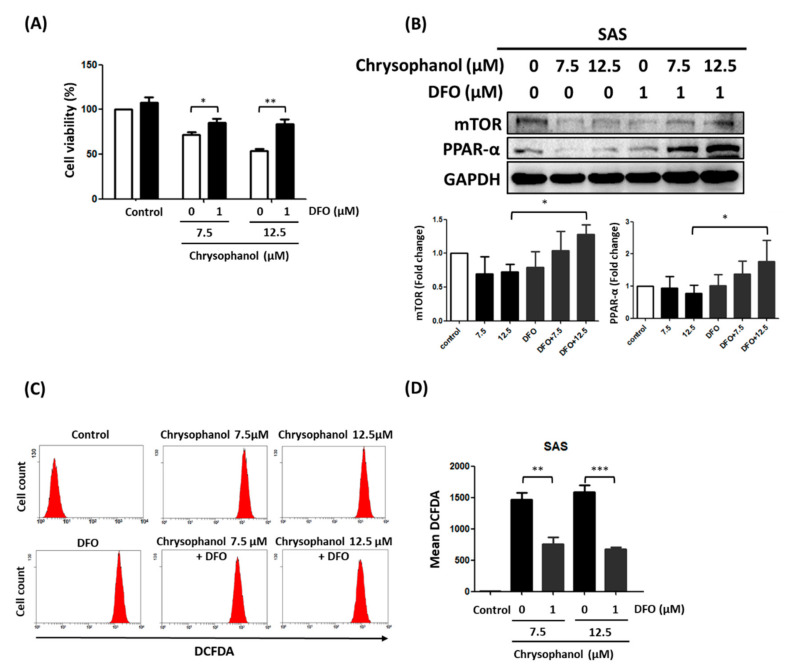
Effect of DFO on cell survival, mTOR/PPAR-α expression, and ROS production in chrysophanol-treated SAS cells. (**A**) WST-1 was used to measure viability of cells in indicated groups. (**B**) Upper panel indicates expression changes of mTOR and PPAR-α proteins in the presence of 0, 7.5, and 12.5 μM chrysophanol with (1 μM) or without DFO treatment. Lower panel indicates quantitative results of assessing specific proteins using ImageJ. (**C**) Changes in ROS levels (with 10 μM DEFDA staining) with 0 (control), 7.5, and 12.5 μM chrysophanol treatment with (1 μM) or without DFO treatment. (**D**) Mean fluorescence intensity was used to measure lipid ROS levels. Three independent replicates were used to calculate standard deviation; *n* = 3. * *p* < 0.05; ** *p* < 0.01; *** *p* < 0.001.

In our previous study, we proposed that chrysophanol-based therapy may decrease oral cancer progression by activating ferroptosis [19]; however, the effect of DFO on the regulation of cell death, mTOR/PPAR-α expression, and ROS accumulation in chrysophanol-treated SAS cells is poorly understood. The results of this study show that 1 μM DFO significantly alleviated the decrease in cell viability in the presence of 12.5 μM chrysophanol, but slightly reversed it in 7.5 μM chrysophanol (Figure 2A). Moreover, 1 μM DFO reversed the downregulation of mTOR and PPAR-α in 12.5 μM chrysophanol, but 7.5 μM chrysophanol slightly reversed it (Figure 2B). Interestingly, 1 μM DFO significantly attenuated chrysophanol-induced ROS accumulation at both 7.5 and 12.5 μM (Figure 2C,D).

**Figure 3:**
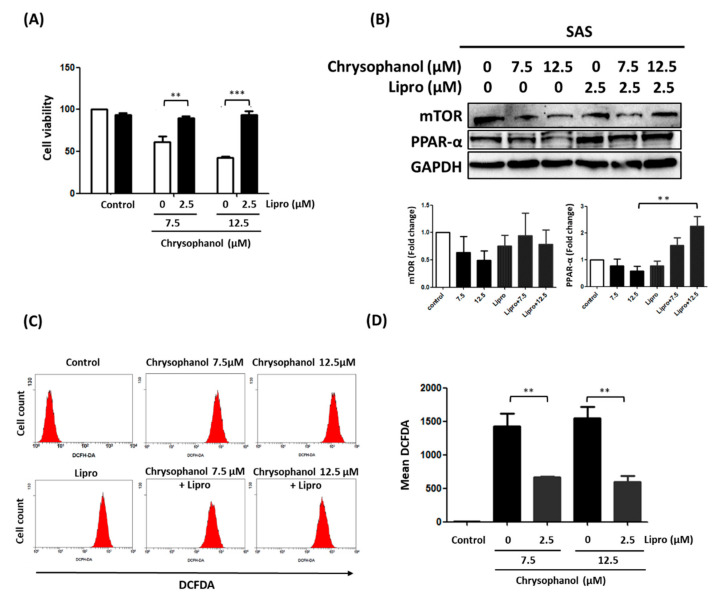
Effect of Lipro on cell survival, mTOR/PPAR-α expression, and ROS production in chrysophanol-treated SAS cells. (**A**) WST-1 was used to measure viability of cells in indicated groups. (**B**) Upper panel indicates expression changes of mTOR and PPAR-α proteins in the presence of 0, 7.5, and 12.5 μM chrysophanol with (2.5 μM) or without Lipro treatment. Lower panel indicates quantitative results of assessing specific proteins using ImageJ. (**C**) Changes in ROS levels (with 10 μM DEFDA staining) with 0 (control), 7.5, and 12.5 μM chrysophanol treatment with (2.5 μM) or without Lipro treatment. (**D**) Mean fluorescence intensity was used to measure lipid ROS levels. Three independent replicates were used to calculate standard deviation; *n* = 3. ** *p* < 0.01; *** *p* < 0.001.

**Figure 4:**
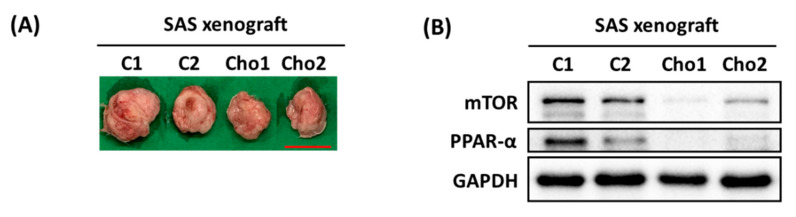
Effect of chrysophanol on SAS-xenograft mouse model. (**A**) Photos show tumors excised after sacrifice on day 30. (**B**) Changes in mTOR and PPAR-α expression in tumors from BALB/cAnN.Cg-Foxn1nu/CrlNarl mice from two groups: control (saline) and chrysophanol 1.67 mg/kg 3 times per week for 4 weeks. C1 and C2 indicate independent mice injected with 5 × 10^6^ cells without chrysophanol treatment (*n* = 2). Cho1 and Cho2 indicate independent mice injected with 5 × 10^6^ cells with 1.67 mg/kg chrysophanol treatment (*n* = 2). Scale bar = 2.5 cm.

Fan et al. reported that the effects of Lipro were more potent than those of daravone or deferoxamine [22]. We studied the pharmacological effect of Lipro on chrysophanol-treated SAS cells by measuring cell viability, mTOR/PPAR-α expression, and ROS levels. The results indicate that Lipro significantly prevented cell death in both 7.5 and 12.5 μM treatment groups (Figure 3A). Conversely, 2.5 μM Lipro reversed the downregulated expression of PPAR with 12.5 μM chrysophanol treatment but had no effect on mTOR expression (Figure 3B). ROS accumulation was also reversed after 2.5 μM Lipro in the presence of 7.5 and 12.5 μM chrysophanol (Figure 3C,D). Further verifying that chrysophanol has an anti-tumor effect in vivo, reduced tumor volume and growth were observed in the group treated with 1.67 mg/kg chrysophanol compared with the control group of SAS-xenograft mice (Figure 4A). The results also show that mTOR and PPAR-α expression was decreased in the chrysophanol-treated group (Figure 4B). These findings suggest that chrysophanol regulates cell death, mTOR/PPAR-α expression, and ROS accumulation in an iron-dependent manner.

## 4. Discussion

Head and neck squamous cell carcinoma (HNSCC) and OSCC are devastating diseases [23,24]. Generally, oral cavity cancers are treated with surgery and chemoradiotherapy (CRT), while pharyngeal and laryngeal cancers are treated with primary CRT [4]. Two immune checkpoint inhibitors, pembrolizumab and nivolumab, have been approved by the FDA as primary treatments for unresectable, recurrent, or metastatic HNSCC [25]. In addition, radiation therapy is one of the most effective treatments for patients with HNC [26]. Importantly, HNSCC develops radiation resistance despite advances in treatment, thus yielding poor survival rates [12]. Radiation causes double-strand breaks in DNA, which kill cancer cells; however, damaged DNA can be effectively repaired to increase radiation resistance [27].

Yu et al. suggested that AKT/mTOR inhibition and G1/G2/M arrest radiosensitize OSCC with AZD2014 [28]. High expression of circular RNA CDR1 has been reported to enhance the viability of OSCC cells in a hypoxic microenvironment by promoting autophagy via the AKT/ERK½/mTOR signaling pathway [29]. A similar study demonstrated that AKT/mTOR-mediated suppression of autophagy-mediated apoptosis by long noncoding RNA CASC9 is associated with tumor progression in OSCCs [30]. Genipin also suppresses OSCC cells via the PI3K/AKT/mTOR signaling pathway [31].

Accumulating evidence indicates that chrysophanol has an anti-tumor effect on various cancer cell lines in different concentrations [8], including human lung cancer A549 cells (50 μM) [32], human liver cancer J5 cells (120 μM) [33], human renal cell carcinoma Caki-2 cells (20 μM) [34], and human colon cancer SNU-C5 cells (120 μM) [35]. The cellular response to ROS-induced lipid peroxidation is manifested in a seamless balance between life and death in response to stress caused by apoptosis, autophagy, or ferroptosis [36]. ROS from the Fenton reaction, which follows lipid peroxidation, causes ferroptosis, which is oxidative cell death caused by iron [37]. In our previous studies, we proposed that the iron chelator DFO reversed the increase in lipid ROS production and decrease in renal cell viability under hypoxia/reoxygenation conditions [18]. However, the effects and mechanisms of action of DFO on OSCC cells are still unclear. Consistent with our findings, Bayeva et al. demonstrated that mTOR modulates the stability of transferrin receptor 1 and alters cellular iron flux by modulating mTOR levels [38]. Similar results have been described, showing that multiple pathways interfere with mTORC1 activation by iron chelation, and iron is necessary for mTORC1 activation [39,40,41]. However, some reports demonstrated that the iron-independent effects of deferoxamine and liproxstatin-1 may be observed in cancer cell survival [22,42]. On the other hand, our novel findings demonstrate that chrysophanol regulates PPAR-α expression in SAS cell lines in an iron-dependent manner. However, the anti-cancer effects of chrysophanol require further examination, especially to determine whether iron is critical to the anti-cancer effects of chrysophanol. Taken together, the results of our study indicate that chrysophanol is a crucial regulator of mTOR/PPAR-α and that modulating its function could help mitigate the carcinogenesis of OSCC cells.

## 5. Conclusions

**Figure 5:**
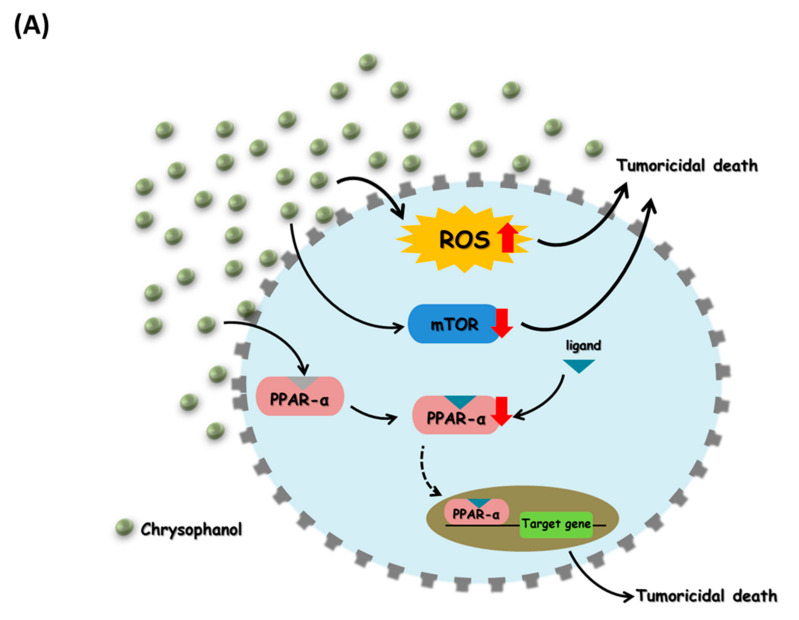

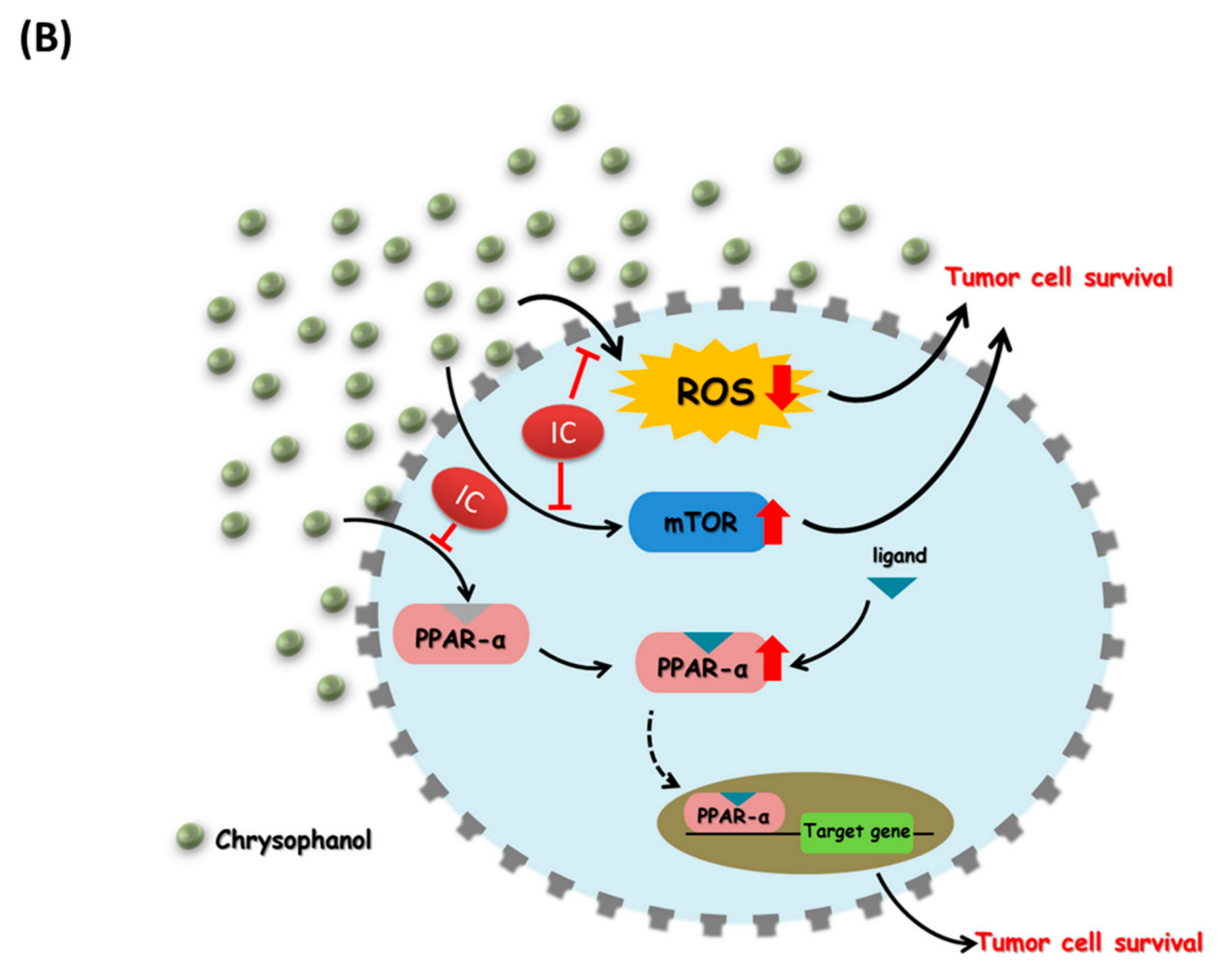
Schematic diagram of chrysophanol and its role in regulating tumoricidal death in SAS cells. (**A**) Chrysophanol-induced tumoricidal death via increased ROS accumulation and decreased mTOR/PPAR-α expression. (**B**) Iron chelator (IC; DFO and Lipro were used in this study) attenuated chrysophanol-induced tumoricidal death and caused tumor cell survival via decreased ROS accumulation and increased mTOR/PPAR-α expression.

These results show that chrysophanol inhibits the growth of oral squamous cell carcinoma cells by modulating mTOR/PPAR-α and ROS accumulation. Iron is thought to play an important role in this process (Figure 5). This study provides a new drug candidate for OSCC treatment. However, even though chrysophanol was able to inhibit tumor cell growth in a cell model, its protective mechanism in vivo needs to be further explored.

## Data Availability

The data used to support the findings of this study are included in the article.

## References

[1] Kamangar F., Dores G.M., Anderson W.F. (2006). Patterns of cancer incidence, mortality, and prevalence across five continents: Defining priorities to reduce cancer disparities in different geographic regions of the world. J. Clin. Oncol..

[2] Chen Y.J., Lin S.C., Kao T., Chang C.S., Hong P.S., Shieh T.M., Chang K.W. (2004). Genome-wide profiling of oral squamous cell carcinoma. J. Pathol..

[3] Ray J.G., Ganguly M., Rao B.S., Mukherjee S., Mahato B., Chaudhuri K. (2013). Clinico-epidemiological profile of oral potentially malignant and malignant conditions among areca nut, tobacco and alcohol users in Eastern India: A hospital based study. J. Oral. Maxillofac. Pathol..

[4] Johnson D.E., Burtness B., Leemans C.R., Lui V.W.Y., Bauman J.E., Grandis J.R. (2020). Head and neck squamous cell carcinoma. Nat. Rev. Dis. Primers.

[5] Warnakulasuriya S. (2010). Living with oral cancer: Epidemiology with particular reference to prevalence and life-style changes that influence survival. Oral. Oncol..

[6] Miguelanez-Medran B.C., Pozo-Kreilinger J.J., Cebrian-Carretero J.L., Martinez-Garcia M.A., Lopez-Sanchez A.F. (2019). Oral squamous cell carcinoma of tongue: Histological risk assessment. A pilot study. Med. Oral Patol. Oral Cir. Bucal..

[7] Su S., Wu J., Gao Y., Luo Y., Yang D., Wang P. (2020). The pharmacological properties of chrysophanol, the recent advances. Biomed. Pharmacother..

[8] Prateeksha, Yusuf M.A., Singh B.N., Sudheer S., Kharwar R.N., Siddiqui S., Abdel-Azeem A.M., Fernandes Fraceto L., Dashora K., Gupta V.K. (2019). Chrysophanol: A Natural Anthraquinone with Multifaceted Biotherapeutic Potential. Biomolecules.

[9] Hsu P.C., Cheng C.F., Hsieh P.C., Chen Y.H., Kuo C.Y., Sytwu H.K. (2020). Chrysophanol Regulates Cell Death, Metastasis, and Reactive Oxygen Species Production in Oral Cancer Cell Lines. Evid.-Based Complement. Alternat. Med..

[10] Chung P.C., Hsieh P.C., Lan C.C., Hsu P.C., Sung M.Y., Lin Y.H., Tzeng I.S., Chiu V., Cheng C.F., Kuo C.Y. (2020). Role of Chrysophanol in Epithelial-Mesenchymal Transition in Oral Cancer Cell Lines via a Wnt-3-Dependent Pathway. Evid.-Based Complement. Alternat. Med..

[11] Hsu P.C., Chen Y.H., Cheng C.F., Kuo C.Y., Sytwu H.K. (2021). Interleukin-6 and Interleukin-8 Regulate STAT3 Activation Migration/Invasion and EMT in Chrysophanol-Treated Oral Cancer Cell Lines. Life.

[12] Machiels J.P., Lambrecht M., Hanin F.X., Duprez T., Gregoire V., Schmitz S., Hamoir M. (2014). Advances in the management of squamous cell carcinoma of the head and neck. F1000Prime Rep..

[13] Ferreira D.M., Neves T.J., Lima L.G.C.A., Alves F.A., Begnami M.D. (2017). Prognostic implications of the phosphatidylinositol 3-kinase/Akt signaling pathway in oral squamous cell carcinoma: Overexpression of p-mTOR indicates an adverse prognosis. Appl. Cancer Res..

[14] Day T.A., Shirai K., O’Brien P.E., Matheus M.G., Godwin K., Sood A.J., Kompelli A., Vick J.A., Martin D., Vitale-Cross L. (2019). Inhibition of mTOR Signaling and Clinical Activity of Rapamycin in Head and Neck Cancer in a Window of Opportunity Trial. Clin. Cancer Res..

[15] Harsha C., Banik K., Ang H.L., Girisa S., Vikkurthi R., Parama D., Rana V., Shabnam B., Khatoon E., Kumar A.P. (2020). Targeting AKT/mTOR in Oral Cancer: Mechanisms and Advances in Clinical Trials. Int. J. Mol. Sci..

[16] Tan Y., Wang M., Yang K., Chi T., Liao Z., Wei P. (2021). PPAR-alpha Modulators as Current and Potential Cancer Treatments. Front. Oncol..

[17] Ligasova A., Vydrzalova M., Burianova R., Bruckova L., Vecerova R., Janostakova A., Koberna K. (2019). A New Sensitive Method for the Detection of Mycoplasmas Using Fluorescence Microscopy. Cells.

[18] Lin C.H., Tseng H.F., Hsieh P.C., Chiu V., Lin T.Y., Lan C.C., Tzeng I.S., Chao H.N., Hsu C.C., Kuo C.Y. (2021). Nephroprotective Role of Chrysophanol in Hypoxia/Reoxygenation-Induced Renal Cell Damage via Apoptosis, ER Stress, and Ferroptosis. Biomedicines.

[19] Lin Y.H., Chiu V., Huang C.Y., Tzeng I.S., Hsieh P.C., Kuo C.Y. (2020). Promotion of Ferroptosis in Oral Cancer Cell Lines by Chrysophanol. Curr. Top. Nutraceutical Res..

[20] Perillo B., Di Donato M., Pezone A., Di Zazzo E., Giovannelli P., Galasso G., Castoria G., Migliaccio A. (2020). ROS in cancer therapy: The bright side of the moon. Exp. Mol. Med..

[21] Weinberg F., Ramnath N., Nagrath D. (2019). Reactive Oxygen Species in the Tumor Microenvironment: An Overview. Cancers.

[22] Fan B.Y., Pang Y.L., Li W.X., Zhao C.X., Zhang Y., Wang X., Ning G.Z., Kong X.H., Liu C., Yao X. (2021). Liproxstatin-1 is an effective inhibitor of oligodendrocyte ferroptosis induced by inhibition of glutathione peroxidase 4. Neural Regen. Res..

[23] Bose P., Brockton N.T., Dort J.C. (2013). Head and neck cancer: From anatomy to biology. Int. J. Cancer.

[24] Chen S.H., Hsiao S.Y., Chang K.Y., Chang J.Y. (2021). New Insights into Oral Squamous Cell Carcinoma: From Clinical Aspects to Molecular Tumorigenesis. Int. J. Mol. Sci..

[25] Kujan O., van Schaijik B., Farah C.S. (2020). Immune Checkpoint Inhibitors in Oral Cavity Squamous Cell Carcinoma and Oral Potentially Malignant Disorders: A Systematic Review. Cancers.

[26] Corvo R. (2007). Evidence-based radiation oncology in head and neck squamous cell carcinoma. Radiother. Oncol..

[27] Hutchinson M.N.D., Mierzwa M., D’Silva N.J. (2020). Radiation resistance in head and neck squamous cell carcinoma: Dire need for an appropriate sensitizer. Oncogene.

[28] Yu C.C., Huang H.B., Hung S.K., Liao H.F., Lee C.C., Lin H.Y., Li S.C., Ho H.C., Hung C.L., Su Y.C. (2016). AZD2014 Radiosensitizes Oral Squamous Cell Carcinoma by Inhibiting AKT/mTOR Axis and Inducing G1/G2/M Cell Cycle Arrest. PLoS ONE.

[29] Gao L., Dou Z.C., Ren W.H., Li S.M., Liang X., Zhi K.Q. (2019). CircCDR1as upregulates autophagy under hypoxia to promote tumor cell survival via AKT/ERK(1/2)/mTOR signaling pathways in oral squamous cell carcinomas. Cell Death Dis..

[30] Yang Y., Chen D., Liu H., Yang K. (2019). Increased expression of lncRNA CASC9 promotes tumor progression by suppressing autophagy-mediated cell apoptosis via the AKT/mTOR pathway in oral squamous cell carcinoma. Cell Death Dis..

[31] Wei M., Wu Y., Liu H., Xie C. (2020). Genipin Induces Autophagy and Suppresses Cell Growth of Oral Squamous Cell Carcinoma via PI3K/AKT/MTOR Pathway. Drug Des. Dev. Ther..

[32] Ni C.H., Yu C.S., Lu H.F., Yang J.S., Huang H.Y., Chen P.Y., Wu S.H., Ip S.W., Chiang S.Y., Lin J.G. (2014). Chrysophanol-induced cell death (necrosis) in human lung cancer A549 cells is mediated through increasing reactive oxygen species and decreasing the level of mitochondrial membrane potential. Environ. Toxicol..

[33] Lu C.C., Yang J.S., Huang A.C., Hsia T.C., Chou S.T., Kuo C.L., Lu H.F., Lee T.H., Wood W.G., Chung J.G. (2010). Chrysophanol induces necrosis through the production of ROS and alteration of ATP levels in J5 human liver cancer cells. Mol. Nutr. Food Res..

[34] Choi J.S. (2016). Chrysophanic Acid Induces Necrosis but not Necroptosis in Human Renal Cell Carcinoma Caki-2 Cells. J. Cancer Prev..

[35] Lee M.S., Cha E.Y., Sul J.Y., Song I.S., Kim J.Y. (2011). Chrysophanic acid blocks proliferation of colon cancer cells by inhibiting EGFR/mTOR pathway. Phytother. Res..

[36] Su L.J., Zhang J.H., Gomez H., Murugan R., Hong X., Xu D., Jiang F., Peng Z.Y. (2019). Reactive Oxygen Species-Induced Lipid Peroxidation in Apoptosis, Autophagy, and Ferroptosis. Oxid. Med. Cell Longev..

[37] Kuang F., Liu J., Tang D., Kang R. (2020). Oxidative Damage and Antioxidant Defense in Ferroptosis. Front. Cell Dev. Biol..

[38] Bayeva M., Khechaduri A., Puig S., Chang H.C., Patial S., Blackshear P.J., Ardehali H. (2012). mTOR regulates cellular iron homeostasis through tristetraprolin. Cell Metab..

[39] Shang C., Zhou H., Liu W., Shen T., Luo Y., Huang S. (2020). Iron chelation inhibits mTORC1 signaling involving activation of AMPK and REDD1/Bnip3 pathways. Oncogene.

[40] Watson A., Lipina C., McArdle H.J., Taylor P.M., Hundal H.S. (2016). Iron depletion suppresses mTORC1-directed signalling in intestinal Caco-2 cells via induction of REDD1. Cell Signal..

[41] Ohyashiki J.H., Kobayashi C., Hamamura R., Okabe S., Tauchi T., Ohyashiki K. (2009). The oral iron chelator deferasirox represses signaling through the mTOR in myeloid leukemia cells by enhancing expression of REDD1. Cancer Sci..

[42] Lee H.J., Lee J., Lee S.K., Lee S.K., Kim E.C. (2007). Differential regulation of iron chelator-induced IL-8 synthesis via MAP kinase and NF-kappaB in immortalized and malignant oral keratinocytes. BMC Cancer.

